# High-Flow Tracheal Oxygen for Tracheostomy Tube Removal in Lung Transplant Recipients

**DOI:** 10.3390/jcm12247566

**Published:** 2023-12-08

**Authors:** Federico Lionello, Gabriella Guarnieri, Giovanna Arcaro, Leonardo Bertagna De Marchi, Beatrice Molena, Cristina Contessa, Annalisa Boscolo, Federico Rea, Paolo Navalesi, Andrea Vianello

**Affiliations:** 1 Department of Cardiac, Thoracic, Vascular Sciences and Public Health, University of Padova, 35131 Padova, Italy; federico.lionello@aopd.veneto.it (F.L.); gabriella.guarnieri@unipd.it (G.G.); giovanna.arcaro@aopd.veneto.it (G.A.); leonardo.bertagnademarchi@aopd.veneto.it (L.B.D.M.); beatrice.molena@unipd.it (B.M.); annalisa.boscolobozza@unipd.it (A.B.); federico.rea@unipd.it (F.R.); 2 Department of Directional Hospital Management, University of Padova, 35131 Padova, Italy; cristina.contessa@aopd.veneto.it; 3 Department of Medicine, University of Padova, 35131 Padova, Italy; paolo.navalesi@unipd.it; 4 Fisiopatologia Respiratoria, Ospedale-Università di Padova, Via Giustiniani, 2, 35128 Padova, Italy

**Keywords:** lung transplant, tracheostomy, high-flow oxygen, mechanical ventilation

## Abstract

(1) Background: Because of a complicated intraoperative course and/or poor recovery of graft function, approximately 15% of lung transplant (LT) recipients require prolonged mechanical ventilation (PMV) and receive a tracheostomy. This prospective study aimed to assess the effect of High-Flow Tracheal Oxygen (HFTO) on tracheostomy tube removal in LT recipients receiving PMV postoperatively. (2) Methods: The clinical course of 14 LT recipients receiving HFTO was prospectively evaluated and compared to that of 13 comparable controls receiving conventional oxygen therapy (COT) via tracheostomy. The study’s primary endpoint was the number of patients whose tracheostomy tube was removed at discharge from an Intermediate Respiratory Care Unit (IRCU). (3) Results: Setting up HFTO proved easy, and it was well tolerated by all the patients. The number of patients whose tracheostomy tube was removed was significantly higher in the HFOT group compared to the COT group [13/14 vs. 6/13 (*p =* 0.0128)]. (4) Conclusions: HFTO is an effective, safe therapy that facilitates tracheostomy tube removal in LT recipients after weaning from PMV.

## 1. Introduction

Because of a complicated intraoperative course and/or poor recovery of graft function, approximately 15% of lung transplant (LT) recipients require prolonged mechanical ventilation (PMV) and receive a tracheostomy [1,2]. Given that the presence of a tracheostomy tube is associated with an increased risk of infections, bleeding, pneumothorax, and tracheal stenosis [3], its removal is an essential step in reducing potential complications and rehabilitating patients recovering from PMV [4].

High-Flow Oxygen through tracheostomy (HFTO) delivers heated, humidified air and oxygen via a dedicated interface at a prescribed fraction of inspired oxygen (FiO_2_) and high flow rates [5]. In 2018, Mitaka et al. described the first attempt to use HFTO during weaning from PMV in patients with restrictive pulmonary dysfunction [6]. Further studies showed uncertain benefits of HFTO compared to conventional (low flow) O_2_ therapy (COT) on physiological and clinical outcomes in critically ill adults who were eligible for decannulation after weaning from mechanical ventilation (MV). Whilst Corley et al. demonstrated that HFTO ameliorates oxygenation in subjects requiring PMV [7], Natalini et al. reported that this intervention did not affect neuro-ventilatory drive, work of breathing, respiratory rate, and gas exchange [8].

In view of its potential benefits on mucociliary transport and mobilization of tracheobronchial secretions [9], and better patient tolerance [10], we hypothesized that HFTO could facilitate tracheostomy tube removal compared to COT. The aim of the study was to assess the efficacy of HFTO on liberation from tracheostomy tube in LT recipients admitted to an Intermediate Respiratory Care Unit (IRCU) for weaning from PMV.

## 2. Methods

This is a single center, prospective cohort study conducted at the IRCU of the University of Padua Medical Centre between 1 November 2019 and 31 August 2022. All the study’s participants signed general consent forms releasing their medical records for review. Ethical approval was waived by the facility’s Institutional Review Committee in view of the fact that all the procedures being performed were part of a hospital’s internal protocol. The study was carried out in accordance with the Declaration of Helsinki of 1975 and the ISHLT Ethics statement.

The clinical course of an unselected group of consecutive double LT recipients admitted to the IRCU for weaning from PMV who received HFTO during the study period (the HFTO group) was prospectively evaluated and compared with that of a population of double LT recipients admitted to the IRCU for weaning between 1 August 2013 and 31 October 2019, who received conventional oxygen therapy via tracheostomy (the COT group). PMV was defined as MV exceeding 21 days for more than 6 h/day [11].

Patients’ baseline demographic and clinical information at admission to the IRCU and clinical/laboratory data on ventilator disconnection were recorded. Arterial blood gas (ABG) data were also recorded at 2 h interval after disconnection from the ventilator.

**Figure 1:**
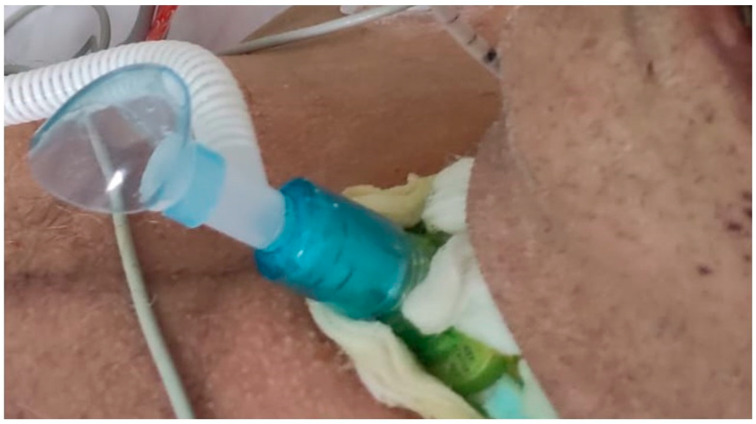
Dedicated tracheostomy interface for High-Flow Tracheal Oxygen.

Weaning from PMV was performed according to a standardized protocol, using gradual daily reduction of pressure support (PS). Immediately after disconnection from the ventilator, patients in the HFTO group received HFTO which was delivered using an AIRVO2 respiratory humidifier (Fisher & Paykel Healthcare, Auckland, New Zealand) with an integrated flow generator able to adjust FIO_2_ (between 0.21 and 1.0) and to deliver an air/oxygen mixture at flow rates of up to 60 L/min. The gas mixture (at 37 °C) was routed through a specific interface for the tracheostomy tube (OPT870, Fisher and Paykel Healthcare, Auckland, New Zealand) (Figure 1).

HFTO was initially used at a 60 L/min gas flow rate and a FIO_2_ of 1.0; it was then adjusted to provide the minimum FIO_2_ necessary to maintain a SaO_2_ ≥ 92%. In the COT group, O_2_ therapy was administered through a Heat Moisture Exchanger (HME) with an integral oxygen inlet connector (Hydro-Trach, Intersurgical, Mirandola, Italy). The gas flow was set to 15 L/min in all the patients; subsequently, O_2_ flow was titrated to maintain a SaO_2_ ≥ 92%. Complications potentially related to treatment including barotraumas, bronchial stenosis and dehiscence of the bronchial anastomosis, were collected in both groups. Moreover, the presence of clinically important CO_2_ retention at a 2 h interval after disconnection was evaluated. Clinically important CO_2_ retention was defined as a rise in PaCO_2_ > 7.5 mmHg [12]. Patients were considered to be ready for decannulation based on standard criteria (Table 1). 

If all criteria were met, the tracheostomy tube was downsized to a tube with an inner diameter of ≤6 mm. The patient was then decannulated after 3 days if arterial blood gases remained satisfactory. 

The study’s primary endpoint was the number of patients who had their tracheostomy tube removed at discharge from IRCU. The study’s secondary endpoints were (a) the number of patients who developed upper-airway respiratory tract infection (URTI) after disconnection, and URTI was diagnosed according to the presence of one or more of the following symptoms or signs: fever, throat irritation or sore throat, and cough [13]; (b) the number of patients who required bronchoscopy-assisted aspiration (BAA) after disconnection; (c) the length of the IRCU stay; and (d) the in-hospital mortality rate. The outcomes were censored on 31 October 2022 for patients still hospitalized on that day. The continuous variables were compared, depending on the normality of the distributions, using the independent unpaired Student’s *t* test or the Mann–Whitney *U* test. Categorical variables were compared, as appropriate, using the chi-squared test or Fisher’s Exact Test. Survival from the time of admission to RICU was calculated using the Kaplan–Meier method; the log rank test was used to compare survival curves between groups.

## 3. Results

Patients’ characteristics and outcomes of the 14 LT recipients who received HFOT were compared with those of 13 patients receiving COT via tracheostomy. Both the patients’ baseline demographic and clinical information and clinical/laboratory data on ventilator disconnection were not significantly different in the two groups, with the exception of the type of immunosuppressive therapy which was modified in 2020 according to a change in the hospital’s internal protocol (Table 2). 

HFTO was simple to set up and well tolerated by all the patients; the incidence of treatment-related complications did not significantly differ between the two groups (Table 3). The number of patients who had their tracheostomy tube removed at discharge from IRCU was significantly higher in the HFOT compared to the COT group (13/14 vs. 6/13 (*p =* 0.0128)). Difficulty swallowing caused failure of tracheostomy tube removal in one patient in the HFTO group. The reasons why patients in the COT group failed tube removal were difficulty swallowing (four cases) and the need for frequent tracheobronchial aspiration (three cases) (Table 3). The number of patients who needed BAA tended to be lower in the HFTO group compared to the COT group (4/14 vs. 8/13 (*p* = 0.1283)). No patient died during hospitalization. At the end of the follow-up period, the log-rank test showed that survival from IRCU admission did not significantly differ between patients in the HFTO and COT group (13.8 ± 3.2 vs. 35.7± 8.9 months (*p* = 0.34)). 

## 4. Discussion

As a widely accepted therapeutic option for patients with a diverse array of end-stage lung disease, LT is currently performed worldwide in more than 4000 patients per year [14]. Despite the significant medical progress achieved in donor management, organ preservation, anesthetic and surgical techniques, perioperative care, postoperative ventilatory strategy, and immunosuppression, LT recipients are still at risk for a variety of complications in the early post-transplantation period, such as infection, rejection, and airway anastomotic dehiscence [1,2,15]. As a result, delay and/or failure of weaning from mechanical ventilation (MV) commonly occurs, due to persisting blood-gas abnormalities and/or muscle fatigue leading to frank hypoventilation and acidosis [16]. In such conditions, administration of High-Flow Nasal Oxygen has been suggested for expediting extubation and providing support to those patients showing persisting hypoxemia [17].

Tracheostomy is commonly used to facilitate weaning from PMV as it is associated with improved comfort, phonation, and oral nutrition, compared with endotracheal intubation; indeed, about 10% of patients requiring more than 3 days of MV are expected to undergo this procedure [18]. However, the presence of a tracheostomy tube has been found to be potentially associated with early and late complications, particularly related to cuff inflation, including infections, bleeding, and tracheal stenosis [19]; for this reason, prompt tracheostomy tube removal is essential in order to reduce the risk of complications and promote participation in rehabilitation programs for patients receiving PMV.

The present study shows that HFTO can be effectively utilized to remove the tracheostomy tube in LT recipients after weaning from PMV. In line with the positive effects of High-Flow Nasal Oxygen (HFNO) on sputum viscosity and cough ability immediately after extubation [20], we hypothesize that HFTO might have better preserved mucociliary clearance in our patients, facilitating secretion elimination and minimizing the need for repeated BAA. Indeed, administration of inhaled gas at core body temperature and 100% relative humidity may preserve mucus rheological properties and prevent the onset of atelectasis [9]. Unlike this, inhalation of dry cold gas during COT may alter the viscosity of respiratory secretions, precipitate bronchoconstriction, reduce ciliary function and mucociliary clearance velocity [21].

Moreover, we hypothesize that HFTO might have prevented swallowing difficulties which can be associated with discomfort caused by breathing under-humidified, cold oxygen [22]. In fact, breathing dry oxygen may cause dryness of the mouth, nose, throat, and respiratory tract, resulting in discomfort and pain that are frequent and multi-factorial in patients admitted to the Intensive Care Unit [23].

Of importance, both frequent tracheobronchial aspiration and inhalation risk are well known reasons why removal of the tracheal cannula can be postponed or even excluded. In particular, a cross-sectional survey of physicians and respiratory therapists with expertise in the management of tracheostomized patients which was conducted to characterize contemporary opinions about tracheostomy decannulation practice showed that cough effectiveness and secretions, as well as level of consciousness and ability to tolerate tracheostomy tube capping, were the most important determinants in the decision to decannulate a tracheostomized patient, whilst swallowing function was judged to be of moderate importance [24].

Of note, HFTO was well tolerated by all the patients. In line with our results, a recent study suggested that heat and humidification of inspired gas may improve comfort and tolerability and alleviate inspiratory effort compared to COT in tracheostomized patients [25]. Moreover, the incidence of barotrauma was negligible, which is especially relevant in LT recipients who are at increased risk for anastomotic leakage [26]. Indeed, one could expect that the onset of air leaks consequent to positive expiratory pressure determined by high-flow oxygen may be facilitated from a “*locus minoris resistentiae*”. 

Given its high tolerability, we hypothesize that HFTO might be also utilized as an effective alternative to Non-Invasive Ventilation (NIV) in subjects showing poor tolerance to the device during the weaning process from a tracheostomy.

The study’s limitations include the small number of patients studied and the use of historical controls, which may have caused a significant bias. It is important to remember, however, that all clinical studies examining patients with rare diseases and/or conditions, such as LT recipients, tend to present these limitations [27]. The study’s long time span is also a potential weakness. Worth mentioning, however, is that we did not make significant changes in pharmacologic therapy and/or supportive care during the study period. As the study was conducted in a single center, the generalizability of its results is, of course, questionable. 

Despite the limitations mentioned above, we believe that our preliminary results are important, in view of the fact that delayed decannulation in LT recipients is associated with a grimmer prognosis and great financial costs. Adequately powered clinical trials are needed to confirm our findings. 

## Figures and Tables

**Table 1:** Standard criteria for tracheostomy decannulation.

Stable clinical condition, in particular the following: ○ Hemodynamic stability; ○ Absence of fever, sepsis, or active infection; ○ Absence of delirium or psychiatric disorders.
Absence of respiratory distress and stable ABG, in particular the following: ○ PaCO_2_ ≤ 45 mm Hg; ○ SaO_2_ ≥ 92% with F_I_O_2_ ≤ 0.3.
Absence of tracheal mucous encumbrance, in particular the following: ○ Tracheal suctioning ≤4 times per 12-h nurse night shift.
Absence of upper-airway abnormalities evaluated through fiberoptic endoscopic examination.
Adequate swallowing evaluated using gag reflex, blue dye, and video fluoroscopy.

**Table 2:** The patients’ baseline demographic and clinical characteristics, clinical and laboratory data on ventilator disconnection. *p*-values refer to differences between HFTO and COT groups. (BGA = Blood Gas Analysis; CHF = Chronic Heart Failure; COPD = Chronic Obstructive Pulmonary Disease; COT = Conventional Oxygen therapy; HFTO = High-Flow Tracheal Oxygen; MI = Myocardial Infarction; NIV = Non-Invasive Ventilation; PaO_2_/FiO_2_ = arterial oxygen tension to inspired oxygen fraction ratio; SaO_2_= arterial oxygen saturation).

	Overall (n = 27)	HFTO Group (n = 14)	COT Group (n = 13)	*p*-Value
Baseline Demographic and Clinical Data				
Age, years	53 (33–64)	51.5 (33–62)	55 (44–64)	0.1815
Female, n (%)	9 (33.3)	5 (35.7)	4 (30.8)	0.9999
Smokers, n (%)	18 (85.7)	9 (75)	9 (100)	0.2285
Body mass index, kg/m^2^	23.5 (16.4–31.2)	23.9 (16.4–31.2)	23.4 (22.1–29.1)	0.8852
Baseline disease, n (%) ○ Idiopathic Pulmonary Fibrosis ○ COPD, emphysema ○ Chronic rejection in LT				
	17 (63)	8 (57.1)	9 (69.2)	0.6945
	8 (29.6)	4 (28.5)	4 (30.8)	0.9999
	2 (7.4)	2 (14.3)	0 (0)	0.4815
Pts with comorbidities, n (%) ○ Metabolic disorder (diabetes, obesity) ○ Hemato-oncology disease ○ Cardiac disease (cardiac arrhythmia, Previous MI, angina pectoris, and/or CHF) ○ Chronic renal failure ○ Psychiatric disorders				
	21 (77.8)	10 (71.4)	11 (84.6)	0.6483
	2 (7.4)	1 (7.1)	1 (7.1)	0.9999
	2 (7.4)	2 (14.3)	0 (0)	0.4814
	2 (7.4)	1 (7.1)	1 (7.1)	0.9999
			
	1 (3.7)	0(0)	1 (7.7)	0.4814
	4 (14.8)	1 (7.1)	3 (23.1)	0.3259
Pre-operative NIV, n (%)	4 (14.8)	1 (7.1)	3 (23.1)	0.3259
Type of immunosuppressive therapy, n (%) ○ Cyclosporine ○ Tacrolimus				
	16 (59.3)	5 (35.7)	11 (84.6)	0.0183
	11 (40.7)	9 (64.3)	2 (15.4)	0.0183
Type of tracheostomy, (surgical/percutaneous)	25/2	13/1	12/1	0.9999
**Clinical, laboratory and BGA data on ventilator disconnection**				
Duration of the weaning protocol, days	3–63	7 (3–29)	12 (3–63)	0.09
Heart rate, beats/min	89.5 (72–113)	88.5 (80–106)	89.5 (72–113)	0.9794
Respiratory rate, breaths/min	17(13–33)	21.5 (17–33)	17 (13–20)	0.0055
Body temperature, (°C)	36.5 (35.6–37.7)	36.5 (35.6–37.7)	36.6 (35.6–37.5)	0.9607
Pts with positive BAL for MDRO, n (%)	17 (63)	7 (50)	10 (76.9)	0.2364
White blood cell count, × 10^9^/L	11.43 (4.98–20.74)	12.77 (4.98–20.74)	9.08 (5.27–18.57)	0.0652
Haemoglobin, g/L	89 (83–106)	89.5 (84–106)	89 (83–98)	0.8840
D-dimer, μg/L	842 (282–4041)	1286 (503–4041)	737 (282–2443)	0.0345
Serum C-reactive protein, mg/dL	30 (2.9–320.0)	31 (3–130)	30 (13–320)	0.6976
Creatinine, μmol/L	70 (25–158)	64 (25–99)	77 (32–158)	0.2641
proBNP, ng/L	463 (45–3359)	405 (199–611)	463 (45–3359)	0.9999
Troponin, ng/L	103 (28–687)	101 (28–251)	375 (62–687)	0.2827
PaO_2_ (O_2_ suppl), mmHg	99.9 (68.7–313.4)	104.0 (68.7–313.4)	91.1 (75.6–195.0)	0.5935
PaCO_2_, mmHg	39.5 (28.6–56.1)	39.5 (28.6–56.0)	40.0 (28.9–56.1)	0.7158
Arterial pH	7.44 (7.36–7.52)	7.44 (7.37–7.50)	7.43 (7.36–7.52)	0.6609
SaO_2_, %	97.1 (93.4–99.3)	97.1 (93.4–99.3)	97.3 (95.3–98.7)	0.9806
PaO_2_/FiO_2_, mmHg	287 (98–522)	269.5 (98–522)	287 (178–487)	0.7709
APACHE II score	10 (6–15)	9.5 (6–14)	10 (8–15)	0.3175

**Table 3:** The patients’ clinical outcomes and treatment-related complications. *p*-values refer to differences between HFTO and COT groups. (BAA = Bronchoscopy-Assisted Aspiration; COT = Conventional Oxygen therapy; HFTO= High-Flow Tracheal Oxygen; IRCU = Intermediate Respiratory Care Unit); URTI = Upper Respiratory Tract Infection.

	Overall (n = 27)	HFTO Group (n = 14)	COT Group (n = 13)	*p*-Value
Patients who underwent decannulation, n (%)	19 (70.4)	13 (92.8)	6 (46.1)	0.0128
Patients who developed URTI, n (%)	4 (14.8)	2 (14.3)	2 (15.4)	0.999
Patients who required BAA ○ Concomitant pneumonia	12 (44.4)	4 (28.6)	8 (61.5)	0.1283
	6 (22.2)	3 (21.4)	3 (23.1)	0.999
Length of IRCU stay, days	21 (3–67)	16 (3–67)	25 (14–59)	0.1565
Death during hospitalization, n (%)	0 (0)	0 (0)	0 (0)	0.999
Treatment-related complications, n (%) ○ Clinically important CO_2_ retention ○ PNX, PNM ○ Bronchial stenosis ○ Dehiscence of bronchial anastomosis				
	0 (0)	0 (0)	0 (0)	0.999
	1 (3.7)	1 (7.1)	0 (0)	0.999
	6 (22.2)	3 (21.4)	3 (21.4)	0.999
	2 (7.4)	1 (7.1)	1 (7.1)	0.999

## Data Availability

The clinical and respiratory function data that support the findings of this study are available at https://intranet.sanita.padova.it at request of the interested party.

## Abbreviations

HFNO	High-Flow Nasal Oxygen
HFTO	High-Flow Tracheal Oxygen
IRCU	Intermediate Respiratory Care Unit
